# Roles of traditional chinese medicine regulating neuroendocrinology on AD treatment

**DOI:** 10.3389/fendo.2022.955618

**Published:** 2022-09-21

**Authors:** Chujun Deng, Huize Chen, Zeyu Meng, Shengxi Meng

**Affiliations:** ^1^ Department of Traditional Chinese Medicine, Shanghai Jiao Tong University Affiliated Sixth People’s Hospital, Shanghai, China; ^2^ The Second Clinical Medical College, Heilongjiang University of Chinese Medicine, Harbin, China

**Keywords:** neuroendocrinology, alzheimer disease, traditional chinese medicine, hypothalamic–pituitary–adrenal axis (HPA) and hypothalamic-pituitary-gonad axis (HPG) axes, insulin, brain gut axis

## Abstract

The incidence of sporadic Alzheimer’s disease (AD) is increasing in recent years. Studies have shown that in addition to some genetic abnormalities, the majority of AD patients has a history of long-term exposure to risk factors. Neuroendocrine related risk factors have been proved to be strongly associated with AD. Long-term hormone disorder can have a direct detrimental effect on the brain by producing an AD-like pathology and result in cognitive decline by impairing neuronal metabolism, plasticity and survival. Traditional Chinese Medicine(TCM) may regulate the complex process of endocrine disorders, and improve metabolic abnormalities, as well as the resulting neuroinflammation and oxidative damage through a variety of pathways. TCM has unique therapeutic advantages in treating early intervention of AD-related neuroendocrine disorders and preventing cognitive decline. This paper reviewed the relationship between neuroendocrine and AD as well as the related TCM treatment and its mechanism. The advantages of TCM intervention on endocrine disorders and some pending problems was also discussed, and new insights for TCM treatment of dementia in the future was provided.

## 1 Introduction

Alzheimer’s disease (AD), a neurodegenerative disease with worldwide increasing prevalence, morbidity and mortality in recent years, is the fifth leading cause of death in adults over 65 years old now (1). The number of reported deaths from AD has increased by more than 145% in the past decade (2). In addition to the sharply rising number of deaths,AD is also burdening patients socially and economically year by year with the social aging process. In recent years, the prevention and treatment of AD has become a hot topic in medical research (3).

Since the neurological damage at late stage of AD is difficult to reverse, the current interest of AD treatment is mainly on early diagnosis, symptomatic treatment, slowing down the development of AD and improving cognitive function. AD as defined in the 2011 NIA-AA guidelines has three stages. Among them, MCI identifies individuals who do not have dementia, but who do have some deficits in cognition (4). 40%-75% of MCI patients may develop AD, and the risk of AD increases with the accumulation of amyloid beta (Aβ) and neurodegeneration. Although AD has a certain genetic tendency, the incidence of AD caused by the combination of multiple etiologies is increasing in recent years (5). Studies have shown that cardiovascular diseases, metabolic disorders, endocrine disorders, anxiety and depression are all risk factors for the development of AD. Early intervention of related risk factors have been proved to have an effect on reducing the risk of AD, even reversing the cognitive loss of MCI patients and delaying the progress of the disease (6, 7).

Previous studies on AD have found that cognitive loss is a cumulative process. Although human brain has a great potential and sufficient cognitive reserve, the onset of sporadic AD is often the result of the joint action of genes and environment, and the continuous accumulation of risk factors often leads to worse outcomes (8). Despite the complex pathogenesis of AD, studies have shown that some endocrine related factors show a significant correlation in the early stage of the disease. The effects of the nervous system and the endocrine system are bidirectional, and both of them jointly regulate brain functions such as nervous system homeostasis, emotion and cognition (9). On the one hand, many metabolic disorders appear before cognitive disorders, so some scholars believe that endocrine disorders are one of the pathogenesis of AD. On the other hand, other scholars believe that due to the regulation effects of the nervous system on endocrine, neurological diseases themselves can also lead to abnormal hormone levels, and some endocrine specific markers have been used for early diagnosis of AD (10). Regulating neuroendocrinology related factors is a promising treatment for people who need to control AD symptoms or reduce the risk of suffering from AD (11).

In traditional Chinese medicine(TCM), Alzheimer’s disease is classified as “forgetfulness”, “fatigue” and “ idiocy”. Based on TCM theory, marrow is an essence for the formation and maintenance of the human body, and is directly related to the functions of the five viscera such as kidney and spleen. Some scholars believe that the marrow can be understood as the connection between endocrine organ and nervous system, and it follows the dynamic balancing process of extraordinary fu organs. Modern studies have also found that the functions of kidney and spleen in TCM are closely related to neuro-endocrine-immune functions. Studies have shown that the deficiency of kidney essence is related to the dysfunction of HPA axis. The role of HPA-mediated BDNF in regulating neuroplasticity is consistent with the traditional notion that the kidney intervenes in brain function by affecting the brain marrow (12). In TCM, the spleen governs transportation and transformation of grain and water and distribution of its essence. Spleen and stomach disorders to cause phlegm and dampness, which blocks the operation of qi and blood. When phlegm and blood stasis stays in the brain for a long time, it is easy to develop AD. Here, the function of the spleen is adapted to the digestive and endocrine functions of the pancreas in modern medicine. Both of them regulate the metabolism of substances and energy in the body. The abnormal metabolism of substances and energy in AD also leads to the deposition of metabolic wastes in the brain, affecting neurological functions and leading to the pathogenesis of AD, which is also consistent with the theory of TCM (13). Therefore, the relevant treatments in traditional medicine are likely to have a good treatment effect on AD. Screening effective TCM treatment by modern technology and further studying of relevant mechanisms can promote TCM modern application.

The course from neuroendocrine disorders to cognitive impairment is a long and cumulative process (14). A variety of factors interact with each other in this process. It is difficult to treat a single target and achieve specific efficacy without having any side effects. This makes TCM have unique advantages over other treatments in AD. Natural chemicals have the characteristics of multi-target and multi-mechanism in living body, and can control the various nerve injury caused by AD. Mechanisms of action of TCM have become the focus of academic attention in recent years (15). Compared with the existing treatment methods of Western medicine for AD, TCM can intervene in early stage of cognitive decline and prevent the progression of AD without causing side effects such as sleep disorders, hallucinations and movement disorders, so it is widely welcomed by most patients (16).

Nerve cells are very sensitive to stress, and the immediate reaction of the nervous and endocrine systems is the survival mechanism during evolution. These organisms respond to changes in the outside world. Chronic stress leads to endocrine disorders, promotes inflammation and oxidative stress, and makes harmful environment continuously act on nerve cells. This also results in the accumulation of toxic substances in the nervous system. Many natural ingredients of TCM can not only restore hormone levels, but also have anti-inflammatory and antioxidant features. This exactly corresponds precisely to the role TCM played in cutting off the vicious cycle in AD (17). Flavonoids are rich in many TCMs. They can reduce neuroinflammatory reactions and regulate oxidative stress related reactions by regulating the autosecretory of nerve cells and the active factors secreted by glial cells. Albiflorin (AL) can reduce AD pathology and improve cognitive impairment by regulating oxidative stress and inflammation in the brain (18). Nobiletin(NOB) can regulate microglia and relieve neuroinflammation (19). Interestingly, TCM can also regulate the level of inflammation in circulation through systemic action, and cut off the harmful communication between peripheral and central organs, thus achieving a fundamental effect in treating AD (20). Studies have shown that some flavonoids themselves have antioxidant and inhibitory effects on amyloid protein aggregation, which is generally related to the position of flavonoid hydroxyl group (21). Abnormal amyloid protein aggregation is also an important pathology of islet dysfunction and AD (22). Flavonoids anthocyanins can enhance insulin sensitivity, weaken insulin resistance at target tissue, and inhibit free aliphatic acid oxidation (23–25). Overlapping mechanisms create preconditions for the dual effects of TCM in endocrine and nervous system (26, 27). In addition to improving the downstream effects of the endocrine axis, some TCM recipes such as ZiBuPiYin Recipe can also ameliorate the imbalance of the hypothalamic-pituitary-adrenal (HPA) axis. On the one hand, it can reduce the hormone level released by the hypothalamus. On the other hand, it can regulate the adrenal gland and peripheral immune organs, and effectively restore the normal hormone level (28).

Apart from regulating endocrine related levels, TCM also has a valid neuroprotective effect (29, 30). For one thing, bioactive compounds in TCM or TCM compound can reduce the synthetize of neurotoxic substances such as Aβ and Tau phosphorylation (31). For another thing, they can promote the clearance of toxic substances by increasing the toxic substance degradation enzyme, improving the blood-brain barrier function and promoting microglia phagocytosis (32, 33). TCM also reduces neuronal apoptosis and loss of neurons caused by excessive autophagy (34, 35). Beyond that, TCM can improve the pathways related to memory storage, and relieve the symptoms of memory loss by protecting the normal energy metabolism of the brain, restoring the structure and function of synapses and restoring neurotransmitter related neurotransmission (36, 37).

TCM can not only reduce memory loss of AD, but also treat mood disorders of AD, such as anxiety and depression, by regulating the stress-related endocrine axis (38). Studies have shown that acupuncture can also restore the circadian rhythm of the HPA axis well, and is an ideal TCM treatment for sleep disorders and emotional anxiety in AD (39). Some Chinese medicines can further regulate the intestinal flora of AD patients and relieve memory disorders through brain-gut axis (40).

## 2 Manuscript formatting

### 2.1 The relationship between neuroendocrine and Alzheimer’s disease

#### 2.1.1 HPA axis and AD

Patients with AD often have HPA axis dysfunction. On the one hand, the release of glucocorticoid (GC), the main product of the HPA axis, promotes changes in metabolic processes in the body by increasing inflammatory response and oxidative stress (41). On the other hand, the brain is highly sensitive to stress, and anxiety enhances amygdala function, alters neural circuits, and leads to structural changes in the frontal lobe and hippocampus (42). These can all be reversed when the physiological level of corticosterone recovers to normal (43). Studies have shown that the elevated cortisol of the AD patient has diagnostic and prognostic value for AD (44).

The hippocampus and frontal lobe are important structures in the brain for learning and memory (45). When the brain is overloaded with glucocorticoids, hippocampal Rab35 (the enzyme required for Tau degradation by endlysosomal sorting) is down-regulated, and Aβ and highly phosphorylated Tau accumulate in large quantities (46). Accumulated toxic substances can eventually lead to cognitive loss by promoting inflammatory response, oxidative stress, energy metabolism disorders and memory related neuron atrophy and synaptic loss (47). GC in the brain is a key regulator of synaptic plasticity and microglial cell activity (48). Excessive release of GC under stress can lead to reduction of glucocorticoid receptor (GR) and down-regulation of its regulated anti-inflammatory effect, promoting neuroinflammation (49). These changes often occur before the onset of AD symptoms. Studies have shown that the HPA axis, circadian, and episodic memory are impaired in the early symptomatic stages of AD (50). Studies have also shown that the application of partial glucocorticoid receptor antagonists can improve neurotransmitter dysfunction and synaptic dysfunction, thus promoting memory storage (51, 52).

#### 2.1.2 Cerebral insulin signal transduction and AD

The insulin signaling pathway itself as well plays an important role in memory and cognition. Insulin receptors(IRs) are widely distributed in the nervus centralis and parts of brain are able to secrete insulin themselves (53). Recent studies have shown that IRs play an important role in the significant links related to AD, including emotion, behavior, cognition, regulation of energy metabolism, regulation of neuronutrition and synaptic plasticity (54). A growing number of clinical studies have also shown that the longer the course of type 2 diabetes (T2D), the higher the risk of getting AD (55). Careful management of cholesterol and glucose from early adulthood can reduce the risk of AD (56). Improving glucose metabolism in MCI patients can reverse cognitive impairment and reduce the risk of developing dementia (57).

Activation of the cerebral insulin signaling pathway begins with the binding of ligand insulin to IR, and subsequent phosphorylation of IRS-1 affects PI3K/AKT signaling. PI3K/AKT signaling is regulated by hormones and connects to a variety of downstream AD-related effects by regulating a variety of transcription factors and cellular functions.PI3K/AKT signaling, which regulates many transcription factors such as CREB and NFκB, is associated with neuronal survival and inflammation. In addition, the following pathways are also involved: autophagy pathways like mTOR; apoptosis pathways like FoxO1, Bax, Bcl-2, JNK, ERK1/2; cerebral vasodilation factors like eNOS and NO. All of these effects can mediate neuron survival and synaptic plasticity, which is a popular pathway related to cognition in recent years (58). PI3K/AKT signaling is also related to pathways that mediate energy metabolism. Various factors that block PI3K/AKT signaling can lead to inactivation of GSK-3β, which further leads to tau phosphorylation and NFT formation. The activation of GSK-3β leads to CDK5 activation through regulation of P25. It is known that these two signal cascades are closely related to abnormal phosphorylation of Tau and play a main role in the pathological process of AD. Under normal circumstances, GSK3β inactivation causes Glut4 to be released from storage vesicles and move to the cell membrane. Hippocampal neurons increase glucose uptake and memory-related activities through this way (59). It has also been confirmed that when AD occurs, inflammation of the nervous system and activation of cellular stress can damage insulin signal transduction and result in brain BBB insulin resistance (60). Insulin resistance affects glucose metabolism and energy homeostasis in the brain, which constitute a vicious cycle and promote the pathological development of AD (61).

Some active factors associated with the insulin pathway are also involved in the development of AD. Glucagon-like peptide-1 (GLP-1) can be produced in the brain and acts like GSK-3β inhibitor (62, 63). GLP-1 activates the GLP-1 receptor signaling pathway and enhances hippocampus learning and memory, promoting neurogenesis, reducing inflammation and apoptosis by activating protein kinase A (PKA), phosphorylating Akt and CREB and reducing Aβ (64, 65). Decreased peripheral and cerebrospinal fluid IGF-1 levels may be a potential marker of cognitive decline and progression in AD (66). Insulin-degrading enzyme (IDE) has a great ability to degrade insulin and Aβ42 (67). IDE acts as an important regulator of Aβ clearance and diabetes, which is associated with neurodegeneration in AD (68).

#### 2.1.3 HPG axis and AD

Being female is second only to advanced age as a risk factor for AD. AD affects more women than men, approaching 2:1 in many countries. Studies have shown that HPG axis changes are an important risk factor for AD. HPG axis hormones include Gonadotropinreception hormone(Gn RH), Luteinizing HRMone (LH), human Chorionic gonadotropin(h CG) and sex hormones. They are extensively involved in neuronal development, structure and brain function.

Studies in animal models and in patients with AD have shown that GnRH administration increases local estrogen levels, protects neural function from amyloid beta (Aβ) neurotoxicity, and prevents cognitive decline (69). Estrogen has been proved to have good neuroprotective effects and to prevent the pathologic development of Alzheimer’s disease (70). On one hand, estrogen has ideal neurotrophic effects. It can reduce neuroinflammation by inhibiting glial inflammatory activation, and thus reduce Aβ accumulation and pathological conformational changes of Tau to prevent memory disorders (71). On the other hand, estrogen, as a regulator of brain energy metabolism, restores normal glucose metabolism and brain mitochondrial function in AD, and significantly improves brain structural abnormalities and cognitive impairment (72, 73).

Recent studies have suggested that lifetime cumulative estrogen exposure may be related to the occurrence of AD. Although most of the brain is local synthetic estrogen, and the added exogenous estrogen therapy by factors such as age, family history, brain health, existing research shows that the menopausal women can improve cognitive function by taking supplementary estrogen. This suggests that individualized assessment is key to the successful prevention of AD in estrogen therapy (74). Similarly, androgens such as testosterone are regulated by the HPG axis. As the physiological level of testosterone decreases with aging in AD, androgen can prevent Aβ plaque formation and reduce Tau phosphorylation (75). The protective effect of testosterone has been proved to be related to the glycogen synthase pathway (76). In addition, LH has also been confirmed to be involved in APP metabolism and Aβ plaque formation in the hippocampus, and reducing LH can improve cognitive impairment (77, 78).

#### 2.1.4 Brain gut axis and AD

The functions of the brain and gastrointestinal system are interlinked with each other. This bi-directional communication involves neuro-endocrine-immune changes and is strongly associated with the onset of AD (79). Studies have shown that improving intestinal flora can improve glucose tolerance, intestinal barrier dysfunction and dyslipidemia in AD model mice. This delayed brain pathological changes in AD model mice in many ways and relieved spatial learning and memory disorders, showing a bright prospect for treating AD (80).

Intestinal microbiome studies in patients with AD suggest that the unique microbiome changes in AD can be a predictor. Studies on the mechanism of AD induced by the microbiome - gut - brain axis changes suggest that intestinal microbes can create an inflammatory environment, promote protein misfolding, and cause inflammation to spread to the brain and cause pathological changes of AD (81). On the other hand, neuroinflammation can also affect the vagus nerve, resulting in intestinal dysbiosis (82). The microbiome - gut - brain axis is also an important link in the metabolism of substances in AD and can serve as an important link between glucolipid metabolism, insulin sensitivity, inflammation and the pathology of AD (83). Microbiome metabolism can also regulate the biosynthesis of neurotransmitters or their precursors, thus affecting the microbiome - gut - brain axis through the neuroendocrine pathway. Tryptophan metabolism regulates brain neurotransmitter signaling through the microbiome - gut - brain axis.

#### 2.1.5 Interaction of neuroendocrine axes in AD

Recent studies have shown that imbalances in various neuroendocrine related systems, such as the HPA axis, HPG axis, insulin and brain-gut axis, can be observed in people with early stage AD, while related hormone therapy has been proved to achieve better results only in the early stages of aging (84, 85). This suggests that the intervention of AD through the neuroendocrine related axis needs to be carried out before the irreversible accumulation of neurotoxic substances, and there is still a large gap in the relevant early drug treatment in western medicine intervention.

**Figure 1:**
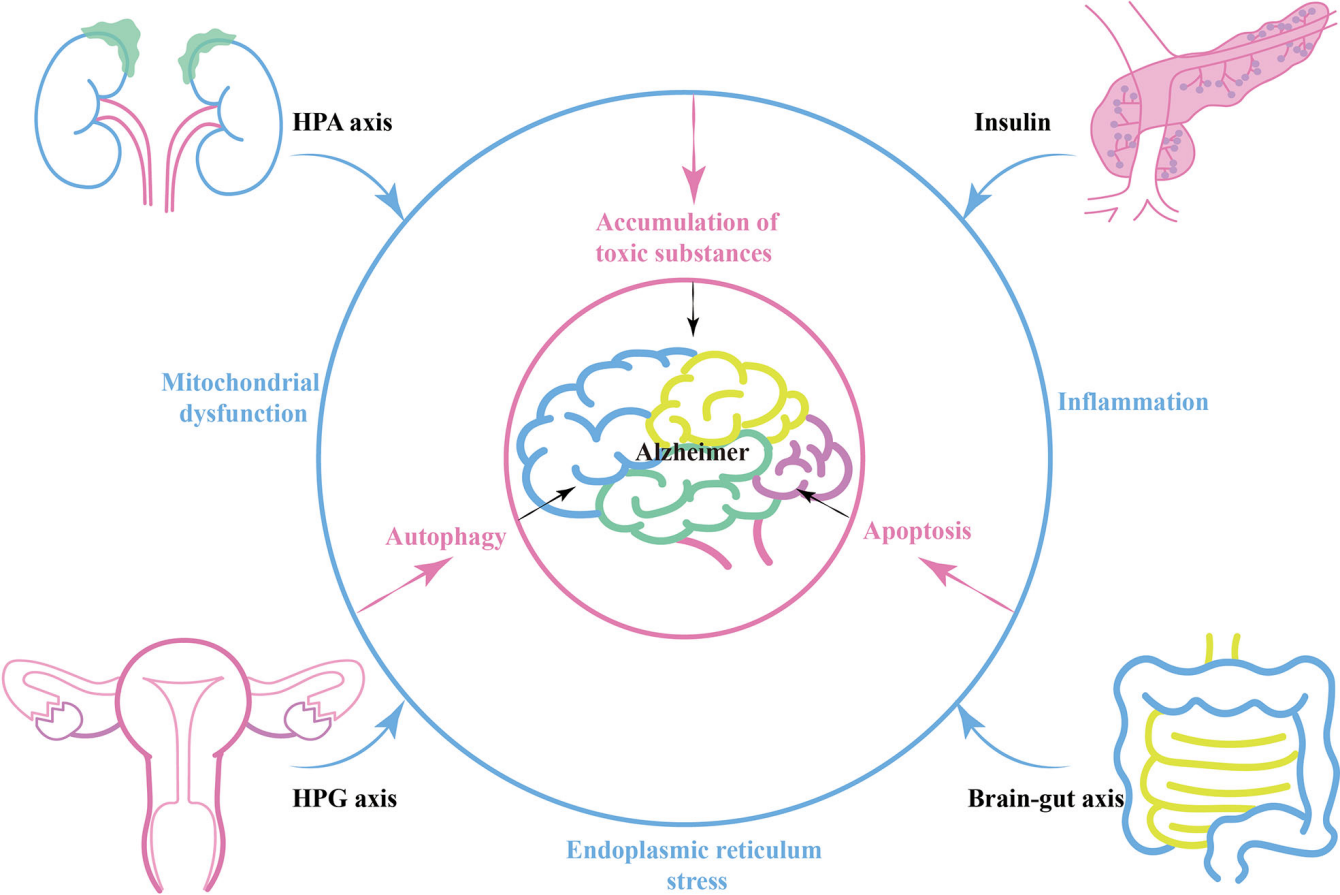
The relationship between neuroendocrine and Alzheimer's disease.

During the process of AD, various neuroendocrine related axes interact and connect with each other through energy metabolism, oxidative stress and inflammation. There is a strong association between diurnal cortisol imbalance and insulin resistance (86). Glucocorticoids can mobilize liver nutrient metabolism and inhibit insulin secretion, and targeting stress-mediated glucocorticoid oversecretion is an effective way to restore the balance of insulin secretion (87). In the brain, hippocampal corticosteroid exposure promotes Tau phosphorylation by activating glycogen synthase kinase 3β (GSK3β), a pathway that intersects with insulin signaling (88, 89). Similarly, the HPG axis intersects with the HPA axis and insulin-related pathways. Estrogen inhibits 11β -hydroxysteroid dehydrogenase type 1, an enzyme involved in the synthesis of bioactive glucocorticoids from its inactive precursor. Estrogen-mediated neuroprotection also interconnects with the IGF-1 signaling pathway (90). Peripherally, either HPA axis imbalance, islet dysfunction or HPA axis imbalance can affect intestinal flora balance and promote chronic expression of peripheral inflammatory markers (91, 92) (Blue circle in **Figure 1**).

Brain-gut axis, peripheral-central immune communication or neuroendocrine related factors can act directly on the brain and alter the internal environment of the nervous system, leading to energy metabolism disorders, oxidative stress, and inflammation in nerve cells (93, 94). In this case, AD-related mitochondrial dysfunction, ER stress, and autophagy related pathways are activated (95). Amyloid precursor protein (APP) secretion increases, while Aβ-degrading enzymes such as enkephalin and insulin-degrading enzymes decrease. This contributes to the accumulation of toxic substances such as Aβ plaques and hyperphosphorylated Tau, resulting in the progression of AD pathology (Pink circle in **Figure 1**).

### 2.2 The neuroendocrine mechanism of TCM treating AD

#### 2.2.1 The neuroendocrine mechanism of TCM regulating HPA axis

##### 2.2.1.1 TCM alleviates the toxic effects of glucocorticoids

Many researches have proved that TCM can regulate the HPA axis, and different medicines can interfere with different sites of glucocorticoid action to improve cognition. Dawn saponin D (ASD) is a widely used TCM monomer that can resist AD, hyperlipidemia, diabetes and osteoporosis. Studies have shown that ASD can reduce the plasma corticosterone and ACTH levels in AD rats, improve memory deficits and anxiety symptoms. ASD shows a good brain protection effect by regulating the HPA axis hormone level (96). Astragaloside (AST) is the main active ingredient extracted from Chinese traditional herb Astragalus membranaceus. Studies have shown that AST can protect hippocampal nerves from glucocorticoid and Aβ25-35 injury, and improve learning and memory disorders. Its mechanism is related to the down-regulation of APP and β secretase mRNA levels. They subsequently caused the decrease of APP and Aβ expression in the hippocampus (97).

**Figure 2:**
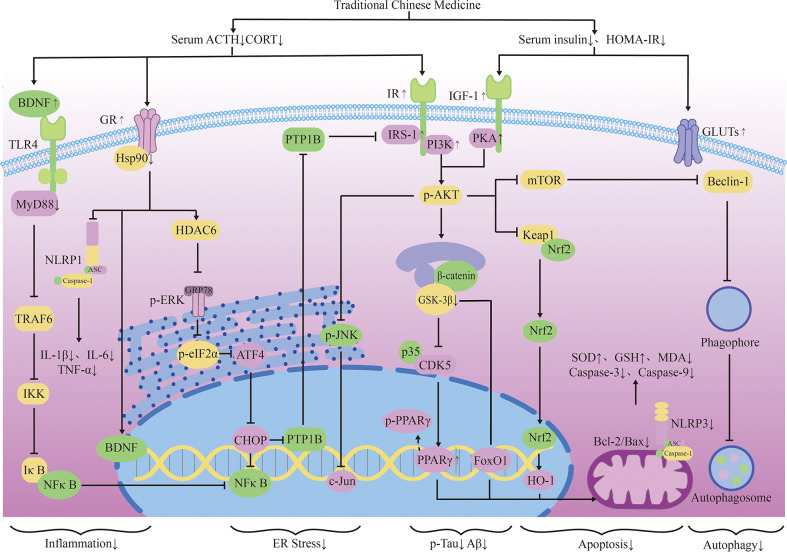
The neuroendocrine mechanism of TCM regulating HPA axis and insulin signal transduction.

Mitochondrial regulation is also an important function of glucocorticoids (98). Chronic cellular stress will activate apoptosis signal transduction, which can promote Aβ production and lead to nerve cell death (99). Some TCMs have been confirmed to protect mitochondrial function and reduce nerve cell apoptosis by fighting ER stress. Ginsenoside Rd(Rd) can inhibit ACTH-induced corticosterone production by blocking the MC2R-cAMP/PKA/CREB pathway in adrenal cortical cells (100). Water extract of ginseng(WEG) can up-regulate the expression of GR and its related functional proteins HDAC6 and Hsp90 to restore mitochondrial function and reduce the succedent expression of ER stress-related proteins. This has followed with reductive nerve cell apoptosis and has shown treatment effects on anxiety and other mental disorders (101). The water extract of Sedum Takesimense (WEST) also has a similar effect (102) (**Table 1** and **Figure 2**).

**Table 1:** TCM treats AD by regulating the mechanism related to HPA axis.

TCM	Method	Animal	Cell	Mechanism	Related pathways	Nerve protection effect	Reference
ASD	Aβ25-35	Rats	/	Serum ACTH↓CORT↓	/	Reduce anxiety symptoms	(92) Y. Wang, et al, 2017
ICA	Aβ25-35	/	HT22	GR↑/BDNF↑	SOD↑、LDH↓、Bcl-2↑、Bax↓、Caspase-3↓	/	(100) C. Tang, et al, 2020
DNLA	12M CUS	Rats	/	Cortex CORT↓/CRF1↑/ACTH↓/GR↑	5-HT↓5-HIAA↓MAO↓、 DA↓DOPAC↓COMT↓	CA2/cortex neural survival↑ Nissl body↑	(102) T. W. Xiong, et al,2021
AMA	/	/	PC12	GR/PLC/PKC↑	INSR/PI3K/AKT/ERK↑、TrkB/Ras/Raf/MEK/ERK↑	Neurogenesis and differentiation↑	(103) L. Cheng, et al, 2021
TEN	CRS	Mice	/	Serum ACTH↓CORT↓ GR↑/TLR4/MyD88/TRAF6/NFκ B	PSD95↑SYN↑	/	(104) H. Wang, et al, 2022
PU	D-gal	Mice	/	GR↑/CREB↑/BDNF↑	/	Neurogenesis and differentiation↑	(101) X. Y. Li, et al, 2017
Rg1	DEX	Mice	/	GR↑/NLRP1↓/ASC↓	IL-1β↓、IL-18↓、Caspase-1↓、Caspase-5↓	Cortex/CA1/CA3 neural survival↑	(105) Y. Zhang, et al, 2017
WEG	corticosterone	/	PC12	GR↑/Hsp90↓/HDAC6↑ CHOP↓、GRP78↓、XBP-1↓	Caspase-12↓、LDH↓ Cytochrome C↓、ICAD↓、Caspase-3↓、Caspase-9↓	Neural survival↑	(97) Y. Jiang, et al, 2015
WEST	corticosterone	/	PC12	CHOP↓、GRP78↓、Bax↓、Bcl-2↑	LDH↓、ROS↓、Cytochrome C↓、Caspase -3↓、Caspase -9↓	Neural survival↑	(98) H. Y. Yun, et al, 2020
TMF	DEX	Mice	/	BACE1 ↓、Aβ↓ Gsk3β↓/p-Tau↓ Raf↑/ERK1/2↑/NF-kB↓	AChE↓ PSD95↑、ADAM10↑、Caspase-3↓	Neuronal apoptosis↓ Neurogenesis and differentiation↑	(106) (107)K. Pakdeepak, et al, 2020
AST	Aβ25-35 DEX	Rats	/	/	APP↓BACE1↓Aβ↓	CA1 neural survival↑	(93) W. Z. Li, et al, 2012

chronic restraint stress, CRS; dexamethasone, DEX; Brain Derived Neurotrophic Factor, BDNF.

The '↑' means up-regulation and '↓' means down-regulation.

##### 2.2.1.2 TCM regulates glucocorticoid receptors and related pathways

Glucocorticoid receptors (GR) in the hippocampus are important for memory formation (108). TCM can regulate toxic Aβ -producing enzymes to protect the brain with elevated stress hormones. They can also promote the production of brain-derived neurotrophic factors(BDNF), which promotes neuronal survival, through glucocorticoid-receptor (GR) related signaling pathways. Icaritin (ICA), the active component of Herba epimedium, has the properties of nerve cell regeneration and anti-apoptosis. Studies have shown that ICA promotes the expression of BDNF and Bcl-2 through GR, thereby inhibiting Bax and caspase-3 and reducing nerve cell apoptosis. ICA can also inhibit the release of lactate dehydrogenase (LDH) and promotes the activity of superoxidase dismutase (SOD), thereby reducing the level of oxidative stress in nerve cells. Its effect can be abolished by GR inhibitors (109). Puerarin (PU) has also been proved to be able to effectively improve learning and memory deficits in mice by promoting GR and BDNF gene expression in the hippocampus and induce neurogenesis (110). Existing studies have reduced the neurotoxic effects of glucocorticoids through selective inhibition of GR, but it has poor application prospects due to the wide distribution and diversity of glucocorticoid receptors. TCM can promote the recovery of normal pathways through the GR pathway. This may be related to the limited activation of the GR can promote cell survival, indicating that the treatment of AD regulated by TCM may be a promising means to reverse brain aging.

Reducing the Chronic activation of the HPA axis can maintain the normal operation of the neurotransmitter system and keep the structure and function of synapses in good condition. Dendrobium officinale alkaloid (DNLA) is an extract of Dendrobium officinale, which has been used in AD, anxiety and depression. DNLA can reduce neuron loss and increase Nissl bodies in the hippocampus CA2 and cortex by attenuating HPA activation and increasing GR expression. Beyond that, DNLA also has reduced the activity of monoamine neurotransmitters and metabolic enzymes elevated by CUS in the brain, and has alleviated the anxiety and depression-like behavior of rats under chronic stress (104). Yokukansan is also a traditional herbal medicine that can counteract the effects of corticosterone, reverse its cytotoxicity to hippocampal neurons and increase neuronal survival in chronic stress rats (111). Its effect of anti-anxiety and alleviation of aggressive symptoms may be mediated by 5-HT receptors (105). Glutamatergic neurological dysfunction is an important link in the pathology of AD, and glutamate receptor is related to nerve excitability. Accumulation of toxic substances in AD will damage receptor function, resulting in calcium imbalance and excitability toxicity. In the end, these will cause autophagy and apoptosis of neurons (103). Tenuifolin (TEN), the main component of Polygala tenuifolia, has neuroprotective performance. TEN can up-regulate the expressions of glucocorticoid receptor, glutamate receptor 1 and synaptic related proteins (107).

Some TCMs can also reduce stress-related inflammation, showing a good effect against inflammatory damage. TEN can reduce serum adrenocorticotropic hormone and corticosterone levels, inhibits Toll-like receptor 4(TLR4)/NFκB mediated inflammation, and regulates the levels of IL-6 and IL-10 in the hippocampus. TEN can also promote the increase of brain-derived neurotrophic factor and myosin kinase B, showing great improvement effect on memory loss caused by chronic stress (107). Ginsenoside Rg1 (Rg1) is the active ingredient in ginseng. Rg1 can protect cerebrovascular endothelium and increases neuronal survival through the GR-ERK signaling pathway, indicating its possible anti-glucocorticoid injury effect (112). In addition, Rg1 decreased NLRP1 inflammasome and ASC and reduced the expression of apoptosis-related factors by activating GR, showing a good cognitive protective effect (113).

It is worth mentioning that in the pathological development of AD, HPA axis and insulin-related pathway show extensive cross-influence. Some Traditional Chinese medicines regulate these cellular endocrinology by affecting their overlapping downstream pathways. JiAmarogentin (AMA) is a dicyclic glycoside isolated from Gentiana rigescens Franch, which has similar effects of nerve growth factor (NGF). Studies have shown that AMA may reduce neuroinflammation and nerve apoptosis through Ras/Raf/ERK and PI3K/AKT signaling pathways regulated by insulin. Interestingly, GR/PLC/PKC signaling pathway has also been found to be involved in the neurogenic effects of AMA. This has indicated possible interactions between GR and insulin-related pathways (114). 5,6,7,4 ‘-tetramethoxylflavanone (TMF) is one of the active ingredients in Chromolaena odorata (l.),and it can reduce BACE1 and PS1 expression to reduce toxic production and synaptic protection. In dexamethasone(DEX) model mice, TMF promoted the expression of proteins associated with neurogenesis, proliferation, differentiation, and maturation through a mechanism similar to AMA (115). Studies have shown that quercetin also decreased serum corticosterone and serum insulin in mice, and increased the expression of GLUT4 in neurons. This indicates its potential to promote neuronal survival by improving energy metabolism, which happens to be an important part of the co-regulation of HPA axis and insulin (116). These studies suggest that TCM intervention in the tandem between glycoskin and insulin pathways may be a better medical option for endocrine-related cognitive risks than mono-acting drugs. Liuwei Dihuang decoction (LW) is a classical TCM prescription, which has potential therapeutic effect on AD. The addition of LW-AFC restores the balance of HPA and HPG axis, enhances the proliferation of spleen cells and corrects the disorder of lymphocyte subsets. It can also regulate abnormal cytokine production in SAMP8 mice, showing superior neuroendocrine immune network regulation over memantine and Donepezil (117) (**Table 1**).

#### 2.2.2 The neuroendocrine mechanism of TCM regulating insulin-related pathways

##### 2.2.2.1 TCM promotes neuronal survival through insulin signaling pathway and related downstream pathways

TCM can reduce oxidative stress. Insulin signaling pathway is related to oxidative stress pathways. TCM can reduce oxidative stress by regulating insulin signaling pathway and activating antioxidant downstream pathways, thus reducing the accumulation of toxic substances and promoting neuronal survival (**Figure 2**). Studies have shown that Lychee seed and Astragalus Membranaceus have beneficial effects on regulating blood glucose, lipids, and anti-oxidation. They can alleviate insulin resistance and oxidative stress in the brain, and show sufficient cognitive benefits (118, 119). Xuefu Zhuyu decoction (XZD) is a tried and tested TCM formula for treating metabolic syndrome, cardiovascular and cerebrovascular diseases. XZD can reduce body weight, insulin resistance, and leptin levels in APP/PS1 mice, thereby improving neuroinflammation and AD-related pathology, demonstrating favorable neuroendocrine regulatory effects (120). In AD patients, insulin receptor substrate 1 (IRS-1) deficiency down-regulates Nrf2/HO-1 signaling, thereby increasing oxidative stress in the brain and ultimately causing nerve cell damage. Thymol is a monoterpene phenol isolated from herb, which has strong neuroprotective effect. Thymol has shown beneficial effects on high fat diet induced cognitive deficits by improving insulin resistance in the hippocampus and activating Nrf2/HO-1 signaling (121). Peganum Harmala (P. Harmala) enhanced Nrf2 through insulin signaling, while reducing lipid peroxidation and adding glutathione (122). Dianxianning (DXN) is a traditional Chinese formula that has been reported to have anti-Alzheimer’s disease activity. Studies have shown that Aβ -induced pathologic features are improved by the insulin-like pathway in transgenic worms treated by DXN (123) (**Table 2** and **Figure 2**).

**Table 2:** TCM promotes neuronal survival through insulin signaling pathway and related downstream pathways.

Effects	TCM	Method	Animal	Cell	Insulin signaling pathway	Related downstream pathways	Toxic substances	Reference
Reduce Oxidative stress	LSE	ICV-STZ HGPD	Rats	/	serum insulin↓、HOMA-IR↓	SOD↑、GSH↑、MDA↓、caspase-3↓	Aβ↓、p-tau↓	(113) Y. Tang, et al, 2018
	APS	APP/PS1 STZ/HFD	Mice	/	serum insulin↓、HOMA-IR↓	SOD↑、GSH↑、MDA↓、caspase-3↓	/	(114) Y. C. Huang, et al, 2017
	Thymol	HFD	Mice	/	p-IRS-1↓/p-AKT↑/GSK-3β↓	Nrf2↑/HO-1↑、SOD↑、MDA↓	Aβ↓、p-tau↓	(116) H. Li, et al. 2017
	P.harmala	AlCl	Rats	/	HI↑/p-IRS-1↓/AKT↑/GSK-3β↓	Nrf2↑、GSH↑、Lipoperoxides↓	Aβ↓、p-tau↓	(117) R. Saleh, et al, 2021
Improve synaptic function	EFAD	HFD	Mice	3T3-L1	p-IRS-1↓/p-AKT↑/p-AMPK↑ TNF-α↓、SOD↑、GSH↑、MDA↓、MMP↑	ACh↑、AChE↓	Aβ↓、p-tau↓	(124) S. Bin Park, et al, 2019
	Hup A	HFD	Mice	/	HI↑/p-AKT↑	ACh↑、AChE↓	BACE1↓ Aβ↓	(125) H. Ying Wang, et al, 2020
	BS	ICV-STZ HFGD	Rats	/	GSK-3β↓、serum insulin↓、HOMA-IR↓ TNF-α↓、IL-1β↓、SOD↑、GSH↑、MDA↓、caspase-3↓	ChE↓ GluR1↑、NR1↑、NR2 A↑	p-Tau↓	(126) A.A.Gomaa,et al,2019
	EC	ICV-STZ HFGD	Rats	/	Gsk3β↓、serum insulin↓、HOMA-IR↓ TNF-α↓、IL-1β↓、SOD↑、GSH↑、MDA↓、caspase-3↓	AChE↓ GluR1↑NR1↑NR2A↑NR2B↑	Aβ↓、p-tau↓	(127) A. A. Gomaa, et al, 2019
Reduce neuronal apoptosis	Genistein	Apoe-/- ob/ob	Mice	/	p-IRS-1↓/p-AKT↓/GSK-3β↓	p-JNK-c-Jun ↓、NGF↑、 BDNF↑	BACE1↓、PS1 ↓ Aβ↓、p-tau↓、	(120) R. Z. Li, et al. 2020 (121) Y. J. Park, et al. 2016
	Q3G	Aβ1-42	Mice	SH-SY5Y	p-IRS-1↓/p-AKT↑/p-AMPK↑/GSK-3β↓/ TNF-α↓、IL-1β↓、IL-6↓、INF-γ↓、IL-10↑、 IL-5↑	p-JNK↓、CREB↑、 BDNF↑	Aβ↓、p-tau↓	(123) M. Xu, et al, 2021
	Magnolol	TgCRND8	Mice	/	p35/CDK5↓/Gsk3β↓ IL-6↓、IL-1β↓、TNF-α↓、CCR2↑	p-JNK ↓/JNK=↓、Bcl-2↑、Caspase-3↓	APP↓BACE1↓ APH1↓PS1↓ Aβ↓、p-tau↓	(128) C. Qu, et al, 2021
	IRN	TgCRND8 Aβ42	Mice	PRHN	IDE↑ IL-6↓、IL-1β↓、TNF-α↓	p-c-Jun↓/c-Jun=↓p-JNK ↓/JNK=↓	APP↓BACE1↓APH1↓PS1↓ Aβ↓、p-Tau↓	(122) H. Q. Li,et al,2019
	EGCG	APP/PS1 HFD	Mice	/	PTP1B↓/GSK3β↓	pERK↓、JNK↓ pEIF2α/ATF4/CHOP↓	ADAM10↑FURIN↑ Aβ↓	(129) M. Ettcheto, et al, 2020
	DNLA	SAMP8	Mice	BV2	Gsk3β↓	pERK↓ ER morphology↑、pEIF2α/ATF4/CHOP↓	Aβ↓、p-tau↓	(130) C. Z. Feng,et al, 2019
	STS	Aβ	/	HT22	NEP↑ 、IDE↑	pEIF2α/ATF4/CHOP↓p-PERK↓	Aβ↓	(131) D. P. Zhang, et al, 2020
	T.chebula	Aβ25–35 HFD	Rats	/	p-Akt↑/GSK-3β↓ TNF-α↓、IL-1 β↓	pFOXO1↑	p-tau↓	(132) S. Park, et al. 2018
	FF	Aβ25–35 HFD	Rats	PC12	p-AKT↑/GSK-3β↓ TNF-α↓、IL-1 β↓	p-FOXO1↓	/	(133) D. S. Kim, et al, 2022
	Silibinin	STZ	Rats	/	IGF-1↑	Bax↓、Bcl-2↑、caspase 3↓	p-tau↓	(118) P. Liu, et al, 2020
Reduce nerve autophagy	BG	ICV-STZ	Mice	/	p-Akt↑/Gsk3β↓ cGMP↑/PKG↑/NF-ĸB↓/BDNF↑、IL-23↓、IL-27↓	AMPK↑、mTOR↓、 beclin-1↓ Wnt3a↑/β-catenin↓/cyclin D1↑	Aβ↓、p-tau↓	(134) M. A. Salem, et al, 2021
Improved energy metabolism	Berberine	STZ HFGD	Rats	/	p-IRS-1↓/PI3K↑/p-AKT↑/GSK-3β↓ PKC↑/IKK↓/NF-κB↓、TNF-α↓、IL-1 β↓	GLUT3↑	BACE1↓、APP↓ Aβ↓	(135) Q. Chen, et al, 2017
	Acteoside	STZ	Rats	/	HI↑/IR↑/IRS1↑	Glu T1↑、Glu T3↑、Glu T4↑ ROS↓、ATP/ADP↑	/	(136) J. Chen, et al. 2021
	Curcumin	APP/PS1	Mice	/	p-IRS-1↓/PI3K↑/p-AKT↑、IR↓/IGF-1R↑	GLUT1↑、GLUT3↑	/	(137) P. Wang, et al, 2017
	P.harmala	AlCl	Rats	/	HI↑/p-IRS-1↓/p-AKT↑/GSK-3β↓、GLP-1↑ ACh↑、AChE↓	GLUT4↑	Aβ↓、p-tau↓	(117) R. Saleh, et al, 2021

High fat diet, HFD; High sugar and fat diet, HFGD; Streptozotocin, STZ; Amyloid cleavage enzyme 1, Bace1; PRHN, primary rat hippocampal neurons; primary cultured rat hippocampal neurons, PCRHN.

The '↑' means up-regulation and '↓' means down-regulation.

TCM can reduce neuronal apoptosis. Neuronal apoptosis is an important part of AD pathology. Neuroinflammation can activate insulin signaling pathway and promote the initiation of apoptosis process. TCM can simultaneously intervene the insulin signaling pathway and its downstream endocrine mechanism about apoptosis, showing a good prospect in the treatment of AD. Silibinin, a polyphenolic flavonoid extracted from silythistle seeds, can significantly inhibit streptozotocin(STZ)-induced neuronal apoptosis, up-regulate insulin signal transduction pathway, and reduce the morphological and structural damage of hippocamal neurons (128). JNK/c-Jun pathway promotes pro-apoptotic protein transcription in AD brain neurons. Treatment with Genistein can increase insulin sensitivity and the expression levels of the neurotrophic factors nerve growth factor (NGF) and brain-derived neurotrophic factor (BDNF) (138). Genistein can also reduce c-Jun N-terminal kinase(JNK) activity and alleviated AD-related pathology in ApoE knockout high fat diet mice (133). Isorhynchophylline (IRN) can inhibit JNK signaling in primary hippocampal neurons treated with Aβ, reducing Aβ production and deposition, and Tau hyperphosphorylation (139). Quercetin-3-o-glucuronide (Q3G), a flavonol Glucuronide, also has similar down-regulation of JNK/c-Jun pathway. In addition, Q3G can also regulate intestinal microbiome ecological imbalance, alleviates cerebral insulin resistance and improves cognitive dysfunction through intestinal brain axis (132). Magnolol also has been proved to have a similar effect on reducing apoptosis and regulating intestinal microbes (140). FoxO1 regulates the transcriptional activity of apoptotic proteins in the nucleus. Studies have also shown that FoxO1 overexpression promotes GSK-3β activity and ERK activity by increasing phosphorylation of GSK-3β (S9), and participates in the process of Tau phosphorylation (131). Forsythia fruit (FF) is a Chinese medicine widely used in treating inflammation. Studies have shown that FF regulates insulin-related pathways and reduces neuronal apoptosis and injury by decreasing FoxO1 activity, thereby improving cognition in rats (129). Luteolin also has a similar effect (130, 141).Endoplasmic reticulum(ER) stress is a major factor of AD nerve apoptosis. ERK activation increases mitochondrial superoxide production and impairs mitochondrial homeostasis. This induces ER stress through the pERK-eIF2α-ATF4-CHOP axis, ultimately leading to neuronal apoptosis (134). ATF4 can guide the transcription of autophagy genes such as Beclin-1, CHOP. The upregulation of CHOP transcription factors is related to neuronal degeneration. The inhibition of insulin signaling pathway caused by increased PTP1B protein is also related to the activation of this pathway. Sodium tanshinone IIA sulfonate (STS) can reduce the level of oxidative stress in SH-SY5Y cells, and reduce ER stress levels by decreasing pERK-eIF2α-ATF4-CHOP axis activation. By protecting ER structure and function, STS has provided cognitive protection (142). Dendrobium officinale can also reduce endoplasmic reticulum stress and Aβ production through a similar pathway (135). EGCG can increase PTP1B protein and reduces activation of this pathway (143) (**Table 2** and **Figure 2**).

TCM can reduce excessive autophagy. Excessive autophagy of nerve cells is a pathogenesis of AD, and the Beclin-1 complex is indispensable in the formation of autophagosome (144). Bergapten (BG) is a TCM with neuroprotective potential. Studies have shown that BG regulates the insulin pathway by stimulating Wnt3a, reduces the activity of GSK-3β, and increases the expression of BDNF. In addition, BG reduces autophagy of nerve cells through the AMPK/mTOR/Beclin-1 pathway, reverses intracerebellar (ICV) STZ injection-induced cognitive impairment, and alleviates AD related pathological manifestations. Huang-Lian-Jie-Du formulae (HLJDD) is a traditional Chinese formula, which has been used in diabetes and Alzheimer’s disease for a long time. HLJDD has been shown to take neuroprotective effects through regulation of glucose and lipid metabolism, up-regulation of autophagy and inhibition of NLRP3 inflammasome signaling pathways (145) (**Table 2**).

TCM can improved energy metabolism. The Glucose transporter protein (GLUTs) family consists of a large group of membrane proteins that transport glucose along a concentration gradient without energy expenditure. GLUT1 and GLUT3 are widely expressed in the central nervous system and are responsible for most glucose uptake and utilization in the brain. Decreased glucose metabolism and energy deficiency associated with GLUTs were observed in AD pathology, which was associated with high Tau phosphorylation and oligomer Aβ production (146). Insulin signaling pathway is closely related to glucose metabolism, and TCM induces GLUTs expression through insulin signaling pathway, alleviates the accumulation of toxic substances induced by energy metabolism disorders, and protects neurotransmitters. Memory impairment has been proved to be related to glucose uptake or metabolism in the medial prefrontal cortex. Berberine can cross the blood-brain barrier and up-regulate GLUT3, promote glucose uptake in the brain, and reduce the expression of amyloid precursor protein and BACE-1, as well as oligomeric Aβ42 production (136, 147, 148). Its cognitive improvement benefit may also be related to the activation of PI3K/Akt/mTOR and MAPK signaling pathways (137, 149). Acteoside can ameliorate STZ-induced oxidative stress and learning and memory impairment caused by GLUT1, GLUT3, and GLUT4 pathways and regulate intracranial metabolism (150). Curcumin has antioxidant and anti-inflammatory properties and can reduce amyloid pathology in AD. Curcumin can increase glucose levels in plasma and brain, and significantly increase GLUT3 and GLUT4 levels (151). Micropositron emission tomography (PET) has shown that curcumin treatment improved cerebral glucose uptake in AD mice (124, 125). Banxia Xiexin decoction (BXD) treatment can not only restore insulin signal transduction, but also increase the expression of glucose transporter 1 (GLUT1) and GLUT3 levels, which had a good effect on the improvement of cognitive ability, synaptic volume and ultrastructure of AD (126) (**Table 2**).

TCM can protect synaptic function. Synaptic loss caused by inflammation and oxidative stress in AD is a major factor related to the progression of the disease. Loss of cholinergic neurons leads to memory and attention deficits, and neurotransmission of glutamate is crucial for synaptic plasticity and neuronal survival (127). TCM can improve chronic stress induced neuroendocrine, neuromicroenvironment, synaptic structure and function disorders through insulin signaling pathway, and relieve patients’ memory loss. Aruncus Dioicus var. kamtschaticus (EFAD) and Huperzine A (Hup A) are both extracts of TCM, which can improve cerebral insulin resistance and cognitive impairment in AD animals. Both of them can increase Ach and decrease AchE and improve synaptic function (152, 153). Polyphenol rich frankincense(BS) gum has been proven to have anti-inflammatory, anticancer and anti-apoptotic effects. BS extracts can significantly enhance glutamate receptor expression (GluR, NR1, NR2 A and NR2B) and reduced Aβ deposition and Tau phosphorylation (154). Cardamom extract (EC) has anti-diabetic, antioxidant and anti-inflammatory properties. In addition to improving cognitive function in rats, EC can also increase the expression of suppressed glutamate receptors (AMPA GluR1 subunit and NMDA receptor subunit NR1, NR2A, NR2B) (155) (**Table 2**).

##### 2.2.2.2 TCM regulates the generation and metabolism of insulin-related enzymes and toxic substances

In the transmission of insulin signaling pathway, the activation of GSK-3β leads to CDK5 activation through the regulation of P25, and both of these signaling cascades are closely related to the abnormal phosphorylation of Tau and play a key role in the pathological process of AD. The total alkaloids from Coptis chinensis Franch (ACF) are widely used in traditional Chinese medicine to treat diabetes and dementia. ACF treatment can significantly increase pIRS, PI3K, and pAkt, restrain GSK3β overactivation, thus reducing Aβ deposition (156). Catechins, proanthocyanidins A1, A2 in Lychee seed and Huperzine A can inhibit hyperphosphorylated Tau through A similar pathway (153, 157). Ginseng has many active components against AD. Pseudoginsenoside-f11 (PF11) is a root and leaf extract of ginseng subspecies. It can ameliorate STZ-induced learning and memory deficits and reduce neuronal loss by regulating insulin signaling and calproteinase I/CDK5 signaling pathways in the hippocampus and thus reducing phosphorylated Tau (158). Dendrobium officinale and ginsenoside Rb1(Rb1) have similar effects (143). CDK5 mediates phosphorylation and inactivation of PPARγ, which has antioxidant and neuroprotective effects (140). Ginsenoside Rg1(Rg1) is one of the main components of ginseng. Studies have shown that Ginsenoside Rg1 can act like CDK5 inhibitor and inhibit PPARγ phosphorylation, thus down-regulating BACE1 and APP expression (159). Notoginsenoside R1 (NTR1), the main active ingredient of ginseng, a well-known traditional Chinese herb, can induce increased levels of peroxisome proliferator-activated receptor γ (PPARγ) and decreases Aβ production (160) (**Figure 2**).

Overexpression of PTP1B negatively regulates insulin signaling by binding to specific phosphorylation residues of IRS-1 (161). Researches have shown that it is also possible that PTP1B itself regulates central nervous system processes associated with neurological diseases. Ferulic Acid(FA) is a phenolic compound can commonly found in a series of plants. Through reducing PTP1B, Ferulic Acid can promote insulin signal transmission, improve spatial memory of middle diabetic rats, increase hippocampal capillary density and reduce aAD-like pathological changes in hippocampus (162, 163) (**Table 3** and **Figure 2**).

**Table 3:** TCM reduces toxic substances through insulin signaling pathway.

TCM	Method	Animal	T	Cell	Mechanism	Toxic substances	Reference
ACF	STZ HFGD	Rats	MWM Y	/	p-IRS-1↓/p-PI3K↑/p-AKT↑/GSK-3β↓	Aβ↓	(152) J. C. Li, et al, 2018
lychee seed	DXM	/	/	HepG2 HT22	p-IRS-1↓/p-PI3K↑/p-AKT↑/GSK-3β↓	p-tau↓	(153) R. Xiong, et al, 2020
Hup A23	HFD	Mice	MWM NOR	/	HI↑/p-AKT↑	BACE1↓ Aβ↓	(125) H. Ying Wang, et al, 2020
PF11	STZ	Rats	NB Y MWM	/	p-IRS-1↓/PI3K↓/p-AKT↑/GSK-3β↓ calpain I↓/CDK5↓	Aβ↓、p-tau↓	(154) L. Zhu, et al, 2021
Rg1	Aβ1−42	/	/	PCRHN	CDK5↓、p-PPARγ↓	APP↓、BACE1↓ Aβ↓	(155) Q. Quan, et al. 2020
NTR1	APP/PS1	Mice	/	N2a-APP695sw	PPARγ↑	Aβ↓	(156) Z. Li, et al, 2015
Magnolol	TgCRND8	Mice	MWM	/	Gsk3β↓/p35/CDK5↓	APP↓BACE1↓APH1↓PS1↓ Aβ↓、p-tau↓	(128) C. Qu, et al, 2021
FA	APP/PS1	Mice	MWM	/	PTP1B↓/p-IRS-1↓/p-Akt↑/GSK3β↓ NFKABAB/	BACE1↓ Aβ↓	(158) N. Y. Wang, et al, 2021
EGCG	APP/PS1 HFD	Mice	MWM ORT	/	PTP1B↓/GSK3β↓	ADAM10↑FURIN↑ Aβ↓	(129) M. Ettcheto, et al, 2020
DNLA	SAMP8	Mice	Y NOR	BV2	Gsk3β↓ Calpain I↓/p35/CDK5↓	Aβ↓、p-tau↓	(130) C. Z. Feng, et al, 2019
Rb1	STZ	Mice	MWM SDT	/	IDE↑、p35/CDK5↓ NMDAR1↑	/	(164) R. Yang, et al, 2020

TAnimal cognitive tests; peroxisome proliferator−activated receptor gamma: PPARγ.

Hippocampal insulin: HI; Nest building: NB; Morris water maze: MWM; Y-maze: Y; Open field test: OFT; Novel object recognition task: NOR; Step-down latency test: SDL; passive avoidance test: PA; Step-Down Test: SDT.

The '↑' means up-regulation and '↓' means down-regulation.

Insulin-degrading enzyme(IDE) and enkephalinase(NEP) have strong ability to degrade insulin and Aβ42, which are associated with neurodegeneration in Alzheimer’s disease (132). EGCG can inhibit NEP expression in astrocytes by activating extracellular signal-regulated kinase (ERK) and phosphoinositol 3-kinase (PI3K) (38, 165). This has promoted Aβ degradation (166). Paeoniflorin (PF) significantly inhibited NO production and secretion of IL-6, IL-1β and tumor necrosis factor-α(TNF-α) in glial cells by up-regulating the NF-κB pathway and the activity of Aβ -degrading enzymes such as IDE and NEP (164). Isorhynchophylline (IRN) has been shown to have significant anti-Alzheimer’s disease activity. In addition to increasing IDE expression, Isorhynchophylline can also inhibit the activation of microglia and astrocytes, reduce tau hyperphosphorylation and neuroinflammation, and improve cognitive impairment, which has a good potential for further development into drug therapy for AD (139). Sarsasapogenin-AA13 can convert pro-inflammatory M1 microglia into anti-inflammatory M2 microglia and increase the expression of IDE and NEP, thus reducing toxic substance deposition (167). A new kind of pectin RP02-1 extracted from the roots of Polygala Tenuifolia, pectin LBP1C-2 purified from the fruits of Lycium berries, Notoginseng Saponin Rg1(Rg1), Rb1 and ginsenoside F1 can up-regulate the expression of Aβ -degrading enzyme in a similar way (106, 152, 168–171) (**Table 4**). LBP1C-2 and Rg1can also decrease the expression of APP, BACE1 and PS1 and increase the expression of α secretase(ADAM10) (172). This leads to the reduction in the APP processing and effectively saves cognitive impairment and neuronal loss (173). Cornel Iridoid Glycoside (CIG) is an active ingredient from cornus officinalis that can also increase ADAM10, NEP and IDE levels in the brain of AD model mice. CIG can also increase the expressions of NGF, BDNF and phosphorylated camp-reactive element binding protein (p-CREB) in the brain of 3×Tg mice, alleviating plaque deposition and cognitive impairment (174). Kai Xin San, a Chinese Herbal formula composed of Radix Ginseng, Poria, Radix Polygalae and Acorus Tatarinowii Rhizome, can induce IDE and result in at least partial remission of hippocampal neuronal injury in rats (175) (**Table 4**).

**Table 4:** TCM increase insulin signaling pathway related toxic degradation enzymes.

TCM	Method	Animal	Cell	Mechanism	Toxic substances	Reference
PF	Aβ25–35	/	C6 glial	NEP↑ 、IDE↑ NOS↓、COX2↓、NO↓ IL-6↓、IL-1β↓、TNF-α↓	Aβ↓	(162) E. J. Cho, et al,2020
DNLA	SAMP8	Mice	BV2	NEP↑ 、IDE↑	Aβ↓、p-Tau↓	(130) C. Z. Feng,et al,2019
NTR1	APP/PS1	Mice	N2a-APP695sw	IDE↑、	Aβ↓	(156) Z. Li, et al,2015
Magnolol	TgCRND8	Mice	/	NEP↑ 、IDE↑ Astrocyte and microglia density↓	APP↓BACE1↓APH1↓PS1↓ Aβ↓、p-Tau↓	(128) C. Qu,et al,2021
IRN	TgCRND8 Aβ42	Mice	PRHN	IDE↑ Astrocyte and microglia density↓、IL-6↓、IL-1β↓、TNF-α↓	APP↓BACE1↓APH1↓ Aβ↓、p-Tau↓	(122) H. Q. Li,et al,2019
AA13	Aβ1-42	Mice	PMA	NEP↑ 、IDE↑ Microglia convert from M1 to M2↑	Aβ↓	(163) C. Huang, et al,2017
RP02-1	/	/	CHO/APPBACE1	NEP↑ 、IDE↑	Aβ↓	(165) H. Zeng, et al,2020
LBP1C-2	/	/	CHO/APPBACE1	IDE↑	BACE1↓ADAM10↑ Aβ↓	(166) L. Zhou, et al,2018
Rg1	Aβ1-42	Rats	/	IDE↑	APP↓BACE1↓PS1↓ADAM10↑ Aβ↓	(169) S. Zhi Liu, et al,2019
F1	APP/PS1 Aβ1-42	Mice	N2aAPP695sw SH-SY5Y	NEP↑ 、IDE↑	Aβ↓	(170) Y. J. Yun, et al, 2022
Rb1	STZ	Mice	/	IDE↑		(164) R. Yang, et al, 2020
DNLA	HMD	Mice	/	NEP↑ 、IDE↑	APP↓BACE1↓PS1↓DNMT1↓ Aβ↓	(167) T. Pi, et al,2021
STS	/	/	SH-SY5Y SH-SY5YAPPsw	NEP↑ 、IDE↑ ROS↓、MDA↓、NO↓、iNOS↓、SOD↑、GSH↑ IL-6↓、IL-1β↓、TNF-α↓	BACE1↓ADAM10↑ Aβ↓	(168) X. Q. Liu, et al,2020
CIG	3×Tg	Mice	/	NEP↑ 、IDE↑ NGF↑、BDNF↑、p-CREB↑	/	(171) C. Yang, et al, 2020
EFAD	HFD	Mice	3T3-L1	IDE↑	Aβ↓、p-tau↓	(124) S. Bin Park, et al, 2019

IDE, Iinsulin degrading enzyme; NGF, nerve growth factor; PMA, Primary microglia and astrocytes.

The '↑' means up-regulation and '↓' means down-regulation.

#### 2.2.3 The neuroendocrine mechanism of TCM regulating HPG axis

Estrogen treatment has been proved to have potential nerve protective effect. But except the nervous system, it may endanger the cardiovascular system and other system. In recent years, some traditional Chinese medicines have been proved to be rich in phytoestrogens, which have a natural similarity with estradiol structure. They can selectively activate estrogen receptors and activate specific neuroprotective effects such as anti-inflammatory, antioxidant and anti-apoptosis, and can be used in AD treatment.

Chinese medicine can reduce toxic substances by activating estrogen receptors. Curcumin can selectively activate estrogen receptor β (Erβ), inhibit IκBα degradation, and reduce BACE1 expression and Aβ level through the NFκB pathway (176). Calycosin, a typical phytoestrogen derived from astragalus membranaceus, binds with estrogen receptors to activate the protein kinase C pathway, reducing oxidative stress and inflammatory response, thereby improving the deposition of toxic substance such as amyloid beta and Tau in the hippocampus of APP/PS1 transgenic mice, and has a good cognitive improvement effect (177).

Some estrogen receptor selective activators have shown great anti-apoptotic effects and can protect nerve cells and synaptic functions. Silibinin, a flavonoid phytoestrogen derived from milk thistle, can improve cognition in AD rats by inhibiting the PI3K-Akt pathway and is a potential drug candidate for the treatment of Alzheimer’s disease (178). Erzhi pills, a classical Chinese medicine prescription that can up-regulate estrogen levels, can also increase the number of Erβ receptors, reduce nerve cell apoptosis and relieve AD pathology by the PI3K-Akt pathway similar to Silibinin (179). Kaempferol can protect PC-12 cells from the apoptotic process by activating estrogen receptors through the ER/ERK-related MAPK signaling pathway (180). Guanghopin alcohol (PTA) is a selective ERβ agonist that can improve oxidative stress and synaptic integrity by enhancing BDNF/TrkB/CREB signaling, and has shown a good neuroprotective effect (181). Interestingly, the quercetin, luteolin, and EGCG mentioned above also have phytoestrogenic properties, which will not be described further in this section due to their similar effects.

**Figure 3:**
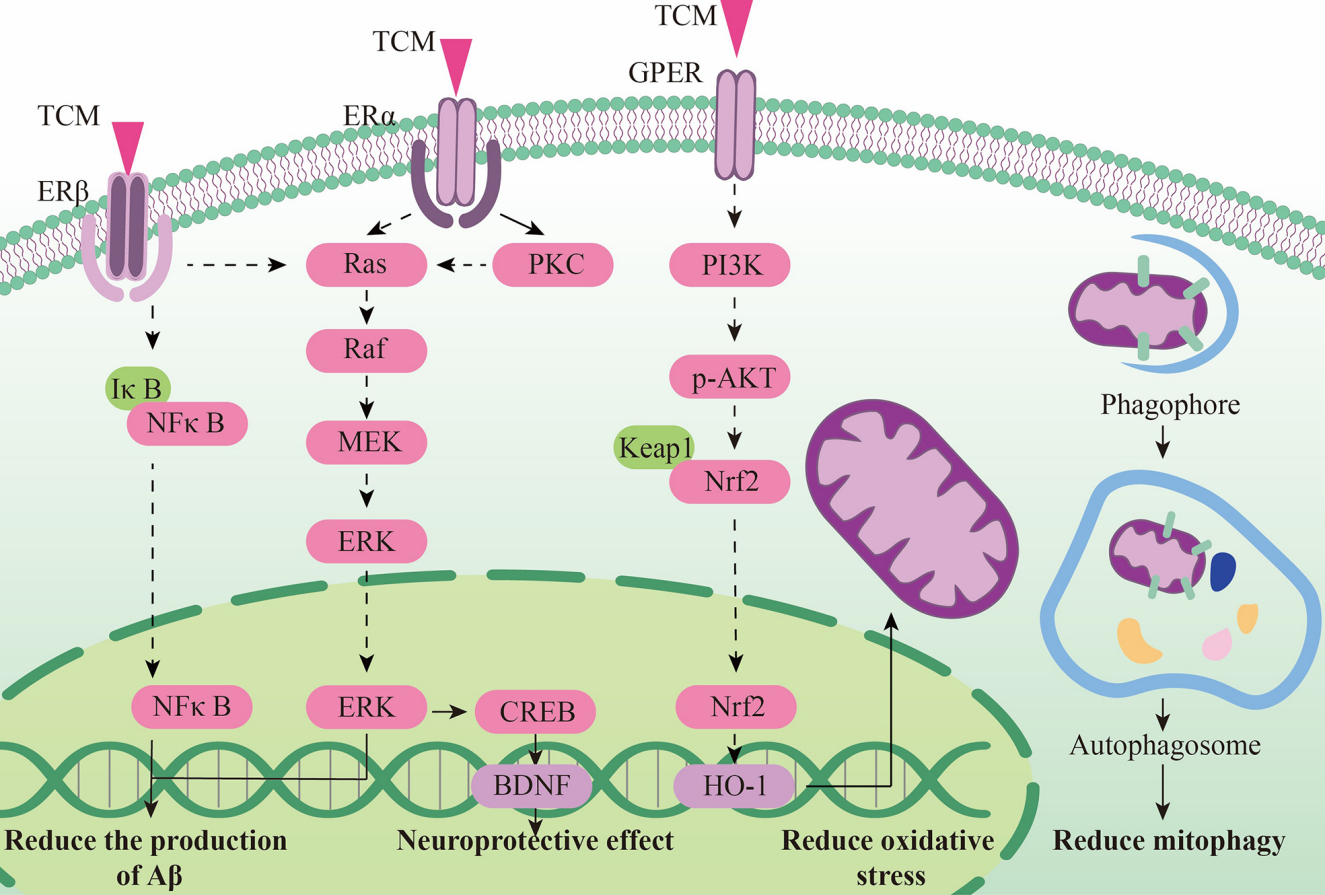
The neuroendocrine mechanism of TCM regulating HPG axis.

In addition, phytoestrogens can also protect endoplasmic reticulum and mitochondrial functions in AD model, showing a good regulation of cell metabolism. Resveratrol (Res) can increase estrogen levels and antioxidant capacity in AD models through the Nrf2/HO-1 signaling pathway, showing a role in regulating mitochondrial function (182). Biochanin A (BCA) is a phytoestrogens isolated from Pratense L. clover that has been commonly used to relieve postmenopausal problems in women. BCA can increase the expression of mitochondrial stability and morphology-related proteins like phosphorylated Drp1, OPA1 and Mfn2, as well as the expression of mitochondrial autophagy related proteins Beclin1, LC3B, Pink1 and Parkin. This shows that BCA can rescue mitochondrial abnormalities, thereby restoring cognitive decline and reducing Aβ deposition and BACE1 expression in oophorectomized APP/PS1 mice (183). Both phytoestrogens α -Zearalanol (α -Zal) and estrogens can effectively reduce the death of AD-like apoptotic neurons, but the side effects of α -ZAl on breast and endometrial tissues are significantly less than that of estrogens. α -ZAl has been proved to have the potential to stabilize ER function by reducing intracellular calcium overload, showing at least partial effects of neuronprotective effect against AD-like apoptosis (184), (**Figure 3**).

There are also studies shows that the activation of estrogen receptors can promote the expression of neurotransmitters and receptors and improve the neurotransmission function. Genistein is a neuroprotective phytoestrogens that can activate estrogen receptor subtypes, regulate NR2B and GLUTamate receptor subunit GluR2 (185), (**Table 5**).

**Table 5:** Mechanisms associated with phytoestrogen treatment with AD.

TCM	Methods	Animal	Cell	Mechanism	Reference
Curcumin	/	/	SH-SY5Y HEK293- APPswe	ERβ↑/NFκB↓ Aβ↓、BACE1↓	(176) Huang P,et al.2020
Resveratrol	SAMP8	Mice	/	ERα/ERβ↑、Nrf2/HO-1↑Aβ↓	(182) Kong D,et al.2019
Genistein	Aβ25-35	/	PRHN	GluR2↑、NR2B↑	(185) Wang Yxiang,et al.2020
Kaempferol	Aβ25-35	/	PC-12	ER/ERK	(180) Zhang N,et al.2020
Biochanin A	APP/PS1 OVX	Mice	/	Beclin1、LC3B↑ p-Drp1、OPA1、Mfn2↑ Aβ↓、BACE1↓	(183) Hou Y,et al.2022
Patchouli alcohol	APP/PS1 PTA	Mice	BV2	ERβ/BDNF/TrkB/CREB	(181) Yan Qying,et al.2022
α-ZAL	Aβ25-35	/	PRHN	GRP78/PERK/CHOP10↓	(184) Yilong D,et al.2017
Calycosin	APP/PS1	Mice	/	PKC	(177) Song L,et al.2017
Silibinin	Aβ1−42	Rats	/	ERER↓/PI3K↓/Akt↓ p-JNK↑ p-p38/p-ERK↓	(178) Xiaoyu S,et al.2018
Erzhi pills	OVXd-gal Aβ1-40	Rats	/	PI3K↑/Akt↑/GSK3β↓ Bad/Bcl-xl/Bcl-2↑ Aβ↓、p-Tau↓	(179) XieY,et al.2021

OVX, Ovariectomize.

The '↑' means up-regulation and '↓' means down-regulation.

#### 2.2.4 The neuroendocrine mechanism of TCM regulating brain gut axis correlation

Most TCM treatments need to be taken orally. Recent studies have shown that some TCM can play an ideal role in the treatment of AD by affecting neuroendocrine factors related to brain-gut axis, such as pro-inflammatory factors, neurotransmitters and active metabolites. Some other TCM require intestinal conversion to become active forms for their neuroprotective effects (186). Some studies have shown that acupuncture can also affect intestinal flora, regulate levels of anti-inflammatory factors, and improve cognition in AD model animals (187). In patients with mild to moderate AD, the combination of abdominal acupoint catenet embedding therapy and Donepezil hydrochloride tablets was superior to monotherapy (188). However, the related mechanism is still unclear and needs further exploration.

Chinese medicine regulates intestinal flora and plays a neuroprotective role by improving inflammatory environment. Gastrodin (Gas) can reduce LPS and pro-inflammatory cytokines in the brain of AD model animals and improve memory by interfering with some intestinal microbiota (189). A novel selenium peptide (SE-PS) with neuroprotective effects obtained from Cordyceps militaris has shown significant protective effects on LPS-induced colon and brain inflammation and oxidative stress and has reduced cognitive impairment in mice by inhibiting the production of pro-inflammatory mediators and malondialdehyde, as well as promoting anti-inflammatory cytokines and the activity of antioxidant enzymes (190). Qisheng Wan Formula (QWF) has been widely used since ancient times to treat patients with amnesia or dementia. QWF has also been proved to improve hippocampal structure in AD rats by inhibiting pro-inflammatory factors and regulating gut microbiota (191).

TCM can regulate the metabolites derived from intestinal microorganisms, improve the metabolism of neurotransmitters and play a neuroprotective role. Xanthoceraside (XAN) is derived from the shell of Xanthoceras sorbifolia Bunge and has anti-AD activity. Although XAN is hardly absorbed by the BBB, it can significantly regulate metabolic disorders including neurotransmitter, amino acid, bile acid and SCFAs metabolism directly or indirectly induced by Aβ1-42 in the intestinal tract. In this way, XAN improves learning and memory deficits in AD rats (192). Similarly, fructose-oligosaccharides from Morinda Officinalis (OMO) can not only maintain the diversity and stability of the gut microbiome, but also have a similar effect in regulating neurotransmitters (193). Through metabonomics correlation analysis, it is beneficial for us to understand the mechanism of action of TCM by studying metabolic pathways related to metabolites derived from intestinal microorganisms. However, specific mechanisms need to be further explored, and relevant studies have been listed in **Table 6**.

**Table 6:** TCM regulates neuroendocrine administration through the brain-gut axis.

TCM	Model	Methods	Memory	Neuroprotective effect	Reference
Gas	Mice	D-gal	Improved	LPS↓,proinflammatory cytokine↓	(189) Fasina OB, et al. 2022
Se-Ps	Mice	LPS	Improved	pro-inflammatory mediator↓, anti-inflammatory cytokines↑	(190) Wu S,et al. 2022
QWF	Rats	Aβ1-42	Improved	pro-inflammatory mediator↓, Improved the hippocampal morphology	(191) Xiong W, et al. 2022
XAN	Rats	Aβ1-42	Improved	Regulation of neurotransmitter, amino acids, bile acids, and SCFAs metabolism	(192) Zhou H, et al. 2022
OMO	Rats	D-gal Aβ1-42	Improved	Improve neurotransmitter synthesis and secretion	(193) Chen D,et al. 2017
Rg1	Mice	3xTg-AD	/	Linoleic acid metabolism,arachidonic acid metabolism,tryptophan metabolism,sphingolipid metabolism	(194) Li G, et al .2019
HAL	Mice	hyoscine	Improved	cholinergic function↑,pro-inflammatory mediator↓	(195) Li SP, et al.2018
Ge	Mice	Aβ1-42	/	Lysophosphatidylcholine metabolism, phenylalanine metabolism	(196) Li J, et al. 2018
BSTSF	Rats	Aβ1-42	Improved	Linoleic acid metabolism,beta-linolenic acid metabolism,glycerophospholipid metabolism,tryptophan metabolism, arginine, proline metabolism	(197) Zhang Z, et al. 2020
HLJDD	Rats	Aβ25-35	/	Methionine metabolism, glutamine metabolism,tryptophan metabolism	(198) Gu X, et al. 2020

The '↑' means up-regulation and '↓' means down-regulation.

## 2.3 Discussion

With the development of modern society, there are more and more sporadic AD patients, many of whom have neuroendocrine related dementia risk factors. Existing western medicine treatment methods for AD mostly focus on a single target to improve neurological function, but due to the complex etiology of AD, a single treatment method often cannot play an ideal role, and some drugs will produce dangerous side effects, which is not well accepted by patients. TCM has unique advantages in treating AD. First of all, as endocrine is A complex organic whole, it is sometimes difficult for drugs with A single target to take into account multiple causes. TCM can simultaneously take into account multiple pathologies of AD in the nervous system and peripheral organs through multi-target action, such as regulating the aggregation of Aβ and pTau in the whole process from generation to metabolism. Regulates inflammation and oxidative stress associated with central and peripheral insulin resistance; Regulate intestinal flora to reduce systemic inflammation, improve the internal environment of the nervous system from the root, and treat AD. Secondly, the use of natural ingredients in TCM have been proved to be safer. Currently western medicine such as Donepezil, lisdamine, galantamine and memantine, are commonly used in AD patients and often have side effects such as headache, dizziness, weight loss, abnormal blood pressure and confusion. None of them are recommended for the prevention of cognitive decline in early stage patients. Many TCM prescriptions, including Liuwei Dihuang decoction, Xuefu Zhuyu decoction and many other recipes mentioned above, have been applied for thousands of years in China. With the development of modern medicine, more evidence-based medical studies have shown satisfying safety and therapeutic benefits of these herbs (199, 200). In view of the objective factors such as the mixture of TCM components in traditional medical treatment, in recent years, researchers have tried to clarify the mechanism of TCM by isolating the active ingredients in formulations or natural Chinese medicinal materials and conducting cell or animal experiments (201). Relevant studies have shown that TCM is an option for patients with AD-related risk factors and mild cognitive decline (202). Some clinical studies have also shown that the combination of TCM and western medicine can have long-term effects on patients with mild cognitive impairment, but larger clinical studies are still needed to enhance the level of evidence (203, 204). Finally, for patients in the early stage of cognitive deficit, timely reduction of various risk factors to promote neurological compensation can greatly improve the quality of life of patients in the later stage. There is a gap in western medicine in this aspect, and Chinese medicine can play a good supplement in this aspect.

In the process of data collection, it was found that some active ingredients of Traditional Chinese medicines such as ginseng, dendrobium, EGCG, silibinin, berberine, curcumin, genistein and so on can regulate various neuroendocrine pathways. This suggests that these drugs have multiple targets in AD, making them more powerful drugs. However, the majority of these studies can not fully explain whether the effects of these drugs on hormone receptors can fully cover their effects, and further experiments are needed. We also found that TCM prescriptions often regulate a variety of neuroendocrine pathways, but the related mechanisms are rarely studied. It was also found that these neuroendocrine related axes often affect mitochondria and endoplasmic reticulum related functions to promote AD susceptibility. More effective drugs and treatments can be found by screening TCM treatments that affect the above related pathways. At present, most of the relevant mechanisms of TCM regulation of the neuroendocrine axis are still not in-depth, such as immune regulation mechanism, metabolism mechanism and other studies need to be further supplemented. Since the neuroendocrine disorders of AD patients vary from person to person, exploring the unique effects of TCMs can provide a basis for personalized treatment.

At present, there are still many deficiencies in the research on the neuroendocrine therapy of AD regulated by TCM. The research on the endocrine regulation mechanism of TCM is still at the surface phenomenon, lacking in-depth mechanism discussion. This is partly due to the use of natural products in TCM, which has the promiscuous characteristics. On the other hand, due to the complexity of endocrine system itself, further research is needed. Due to the differences between animal endocrine physiology and human neuroendocrine physiology, human neuroendocrine physiology has unique characteristics. Although existing animal research models can represent certain characteristics of AD to a certain extent, for sporadic AD with mixed etiology, a model closer to the actual situation still needs to be developed. Although TCM has been used in China and other Asian countries for thousands of years, in the era of modern medicine, more standardized and larger scale clinical studies are necessary to establish widely accepted and recommended TCM treatment plans for dementia.

## Author contributions

CD, HC, and ZM have contributed equally to this work and share first authorship. All authors contributed to the article and approved the submitted version.

## Funding

This work was supported by Project of COVID-19 Emergency Response Project of Shanghai Sixth People’s Hospital in 2022(ynxg202218), Project of Shanghai Science and Technology Commission (19401970600) and Project of Shanghai Science and Technology Commission (19401932500), and Shanghai will further accelerate the 3-year action plan for the development of TCM (2018–2020) for major clinical research on TCM (ZY (2018–2020)-CCCX-4010), the Innovation Fund of Integrated Traditional Chinese and Western Medicine, School of Medicine, Shanghai Jiao Tong University (18zxy002), the 2019 Teacher Training and Development Project of Medical School of Shanghai Jiao Tong University (JFXM201909), and the Experimental Project of Scientific and Technological Innovation for College Students of Heilongjiang University of Traditional Chinese Medicine (16041200019).

## Conflict of interest

The authors declare that the research was conducted in the absence of any commercial or financial relationships that could be construed as a potential conflict of interest.

## Publisher’s note

All claims expressed in this article are solely those of the authors and do not necessarily represent those of their affiliated organizations, or those of the publisher, the editors and the reviewers. Any product that may be evaluated in this article, or claim that may be made by its manufacturer, is not guaranteed or endorsed by the publisher.
